# High Infection Attack Rate after SARS-CoV-2 Delta Surge, Chattogram, Bangladesh

**DOI:** 10.3201/eid2802.212417

**Published:** 2022-02

**Authors:** Sonia T. Hegde, Taufiqur Rahman Bhuiyan, Marjahan Akhtar, Taufiqul Islam, Juan Dent Hulse, Zahid Hasan Khan, Ishtiakul Islam Khan, Shakeel Ahmed, Mamunur Rashid, Rumana Rashid, Emily S. Gurley, Tahmina Shirin, Ashraful Islam Khan, Andrew S. Azman, Firdausi Qadri

**Affiliations:** Johns Hopkins Bloomberg School of Public Health, Baltimore, MD, USA (S.T. Hegde, J.Dent Hulse, E.S. Gurley, A.S. Azman);; International Centre for Diarrhoeal Disease Research, Bangladesh (T.R. Bhuiyan, M. Akhtar, T. Islam, Z.H. Khan, I.I. Khan, A.I. Khan, F. Qadri);; Bangladesh Institute of Tropical and Infectious Diseases, Chattogram, Bangladesh (S. Ahmed, M. Rashid, R. Rashid);; Institute of Epidemiology, Disease Control and Research, Dhaka, Bangladesh (T. Shirin);; Institute of Global Health, University of Geneva, Geneva, Switzerland (A.S. Azman)

**Keywords:** COVID-19, Delta variant, coronavirus disease, severe acute respiratory syndrome coronavirus 2, SARS-CoV-2, attack rate, seroepidemiologic studies, respiratory infections, zoonoses, Bangladesh

To the Editor: After an initial serosurvey (*1*) to understand the prevalence of total antibodies to severe acute respiratory syndrome coronavirus 2 (SARS-CoV-2) in residents of the Sitakunda subdistrict was completed, a large epidemic wave hit the area, and nearly all publicly available samples genotyped via GISAID (https://www.gisaid.org) were the SARS-CoV-2 Delta variant (*2*,*3*). Of the total confirmed infections during the entire pandemic from the Chattogram District, 48.4% (48,253) were reported June 14–August 31, 2021. During September 21–October 9, 2021, we revisited all enrolled households and collected blood from 84% (1,938/2,307) of those tested in our initial serosurvey (Appendix Figure).

We tested 721 of the initially seronegative participants who agreed to a second blood draw using the same Wantai total Ab receptor-binding domain assay and found that 68% (492/721) had seroconverted in the approximately 3-month period between survey rounds (Appendix Table 1). Participation in the second round was not associated with serostatus in the first round. Among seropositive participants, 87 (18%) had received >1 dose of SARS-CoV-2 vaccine, and 28.3% (140/492) of those who seroconverted reported having had a sudden onset of >1 coronavirus disease–related symptom since the first serosurvey. Assuming no seroreversion between rounds, 88.2% (1,709/1,938) of participants providing blood in both rounds were seropositive by the second serosurvey. Using our previous methods (*1*), we estimated an adjusted seroprevalence after the Delta wave of 88.2% (95% CrI 85.4%–90.8%) for all participants and 87.9% (95% CrI 85.2%–90.6%) when including only unvaccinated participants (Appendix Table 2). Seroprevalence among children 1–9 years of age remained significantly lower when compared with 25–34 year olds (28% reduced risk for 1–4 and 16% for 5–9 year age groups; p<0.00001), unlike other age groups (Appendix Table 2). Mirroring evidence from around the world, the Delta variant led to a significant increase in SARS-CoV-2 transmission in Bangladesh, leaving the vast majority of people with detectable serum antibodies. 

Appendix Additional information on serosurvey of SARS-CoV-2 in Chattogram, Bangladesh
