# Supplementary material for: High Infection Attack Rate after SARS-CoV-2 Delta Surge, Chattogram, Bangladesh
Source: Emerg Infect Dis. 2022 Feb;28(2):492–3. doi: 10.3201/eid2802.212417 (PMC8798703; doi:10.3201/eid2802.212417)
Supplement: Appendix — Additional information on serosurvey of SARS-CoV-2 in Chattogram, Bangladesh [file 21-2417-Techapp-s1.pdf]

Article DOI: https://doi.org/10.3201/eid2802.212417

High Infection Attack Rate after SARS-CoV-2 Delta Surge, Chattogram, Bangladesh

Appendix

Appendix Table 1. Overview of participants enrolled and tested in serosurvey rounds 1 and 2. Only participants who were seronegative in round 1 were tested in round 2.*

	Round 1			Round 2			
Variable	Participants tested	Positive	Negative	R1 participants providing blood sample for R2	Participants tested	Positive	Negative
Age, y†							
1–4	90	53 (58.9)	37 (41.1)	70	42	16 (38.1)	26 (61.9)
5–9	174	103 (59.2)	71 (40.8)	146	86	49 (57.0)	37 (43.0)
10–14	258	118 (45.7)	140 (54.3)	226	103	80 (77.7)	23 (22.3)
15–24	482	177 (36.7)	305 (63.3)	405	147	97 (66.0)	50 (34.0)
25–34	381	123 (32.3)	258 (67.7)	311	101	68 (67.3)	33 (32.7)
35–44	325	100 (30.8)	225 (69.2)	280	83	64 (77.1)	19 (22.9)
45–54	250	70 (28.0)	180 (72.0)	216	59	42 (71.2)	17 (28.8)
55–64	208	76 (36.5)	132 (63.5)	174	65	50 (76.9)	15 (23.1)
65–100	139	44 (31.7)	95 (68.3)	110	35	26 (74.3)	9 (25.7)
Sex							
M	1072	690 (64.4)	382 (39.0)	910	313	216 (69.0)	97 (31.0)
F	1235	753 (61.0)	482 (16.7)	1,028	408	276 (67.6)	132 (32.4)
Overall	2,307	1,443 (62.5)	864 (37.5)	1,938	721	492 (68.2)	229 (31.8)

*R1, round 1; R2, round 2

Appendix Table 2. Overview of SARS-CoV-2 seropositivity, seroprevalence, and relative risk seropositivity in Sitakunda Upazila in the second serosurvey after the Delta wave. Adjusted estimates account for sex, age, household clustering, and test performance among all vaccinated and unvaccinated participants*

Variable	Observations	Positive	Negative	Seroprevalence (95% CI)	Relative risk (95% CI)
Age, y†					
1–4	70	44 (62.9)	26 (37.1)	67.1 (55.8–77.4)	0.72 (0.58–0.84)
5–9	146	109 (74.7)	37 (25.3)	77.6 (70.0–84.6)	0.84 (0.75–0.93)
10–14	226	203 (89.8)	23 (10.2)	92.8 (88.1–96.7)	1.03 (0.97–1.09)
15–24	405	355 (87.7)	50 (12.3)	90.4 (86.5–94.1)	1.00 (0.94–1.06)
25–34	311	278 (89.4)	33 (10.6)	90.3 (86.7–93.4)	Referent
35–44	280	261 (93.2)	19 (6.8)	94.9 (91.6–97.6)	1.06 (1.01–1.11)
45–54	216	199 (92.1)	17 (7.9)	93.5 (89.1–97.1)	1.04 (0.98–1.10)
55–64	174	159 (91.4)	15 (8.6)	93.6 (88.6–97.7)	1.04 (0.98–1.10)
65–100	110	101 (91.8)	9 (8.2)	93.8 (88.9–97.5)	1.05 (0.98–1.11)
Sex					
M	910	813 (89.3)	97 (10.7)	90.0 (86.7–93.1)	1.04 (1.00–1.07)
F	1,028	896 (87.2)	132 (12.8)	86.2 (83.0–89.2)	Referent
Overall	1,938	1,709 (88.2)	229 (11.8)	88.2 (85.4–90.8)	NA

*CrI, credible interval; NA, not available
†Age of participants at baseline (first serosurvey).

Appendix Table 3. Alternative estimates of seropositivity and seroprevalence assuming all seronegative participants in the first serosurvey who did not participate in the second round remained seronegative. Using this approach, we estimate a lower bound adjusted seroprevalence after the Delta wave of 83.5% (95% CrI 80.7%–86.3%), 83.1% (95% CrI 80.2%–86.0%) when including only unvaccinated participants.

Variable	Observations	Positive	Negative	Seroprevalence (95% CrI)	Relative risk (95% CrI)
Age, y†					
1–4	90	53 (58.9)	37 (41.1)	60.8 (50.7–70.5)	0.69 (0.57–0.81)
5–9	174	120 (69.0)	54 (31.0)	72.0 (64.7–79.0)	0.83 (0.74–0.91)
10–14	258	220 (85.3)	38 (14.7)	87.6 (82.4–92.3)	1.02 (0.95–1.08)
15–24	482	402 (83.4)	80 (16.6)	85.7 (81.7–89.6)	0.99 (0.94–1.05)
25–34	381	326 (85.6)	55 (14.4)	86.3 (82.3–89.9)	Referent
35–44	325	289 (88.9)	36 (11.1)	90.8 (87.0–94.3)	1.06 (1.00–1.11)
45–54	250	222 (88.8)	28 (11.2)	90.0 (85.4–94.1)	1.05 (0.98–1.11)
55–64	208	182 (87.5)	26 (12.5)	89.9 (84.6–94.5)	1.04 (0.98–1.11)
65–100	139	121 (87.1)	18 (12.9)	84.7 (84.3–94.6)	1.04 (0.97–1.11)
Sex					
M	1072	906 (84.5)	166 (15.5)	84.9 (81.5–88.0)	1.02 (0.99–1.06)
F	1235	1,029 (83.3)	206 (16.7)	82.2 (79.0–85.5)	Referent
Overall	2,307	1,935 (83.9)	372 (16.1)	83.5 (80.7–86.3)	NA

*CrI, credible interval; NA, not available
†Age of participants at baseline (first serosurvey).

Appendix Table 4. Alternative estimates of seropositivity and seroprevalence assuming all seronegative participants in the first serosurvey who did not participate in the second round seroconverted. Using this approach, we estimate an upper bound adjusted seroprevalence after the Delta wave of 90.4% (95% CrI 88.1%–92.6%); and 90.3% (95% CrI 88.0%–92.5%) when including only unvaccinated participants.

Variable	Observations	Positive	Negative	Seroprevalence (95% CrI)	Relative risk (95% CrI)
Age, y†					
1–4	90	64 (71.1)	26 (28.9)	74.9 (65.8–83.2)	0.79 (0.69–0.89)
5–9	174	137 (78.7)	37 (21.3)	81.3 (74.6–87.3)	0.87 (0.79–0.94)
10–14	258	235 (91.1)	23 (8.9)	93.7 (89.7–97.1)	1.02 (0.97–1.07)
15–24	482	432 (89.6)	50 (10.4)	92.3 (89.0–95.2)	1.00 (0.96–1.05)
25–34	381	348 (91.3)	33 (8.7)	92.0 (89.0–94.6)	Referent
35–44	325	306 (94.2)	19 (5.8)	95.8 (93.1–98.1)	1.05 (1.01–1.09)
45–54	250	233 (93.2)	17 (6.8)	94.6 (90.8–97.7)	1.03 (0.98–1.08)
55–64	208	193 (92.8)	15 (7.2)	95.0 (90.8–98.3)	1.04 (0.98–1.09)
65–100	139	130 (93.5)	9 (6.5)	95.2 (91.2–98.1)	1.04 (0.99–1.09)
Sex					
M	1,072	975 (91.0)	97 (9.0)	91.9 (89.2–94.4)	1.03 (1.00–1.06)
F	1,235	1,103 (89.3)	132 (10.7)	88.9 (86.1–91.4)	Referent
Overall	2,307	2,078 (90.1)	229 (9.9)	90.4 (88.1–92.6)	NA

*CrI, credible interval; NA, not available
†Age of participants at baseline (first serosurvey).

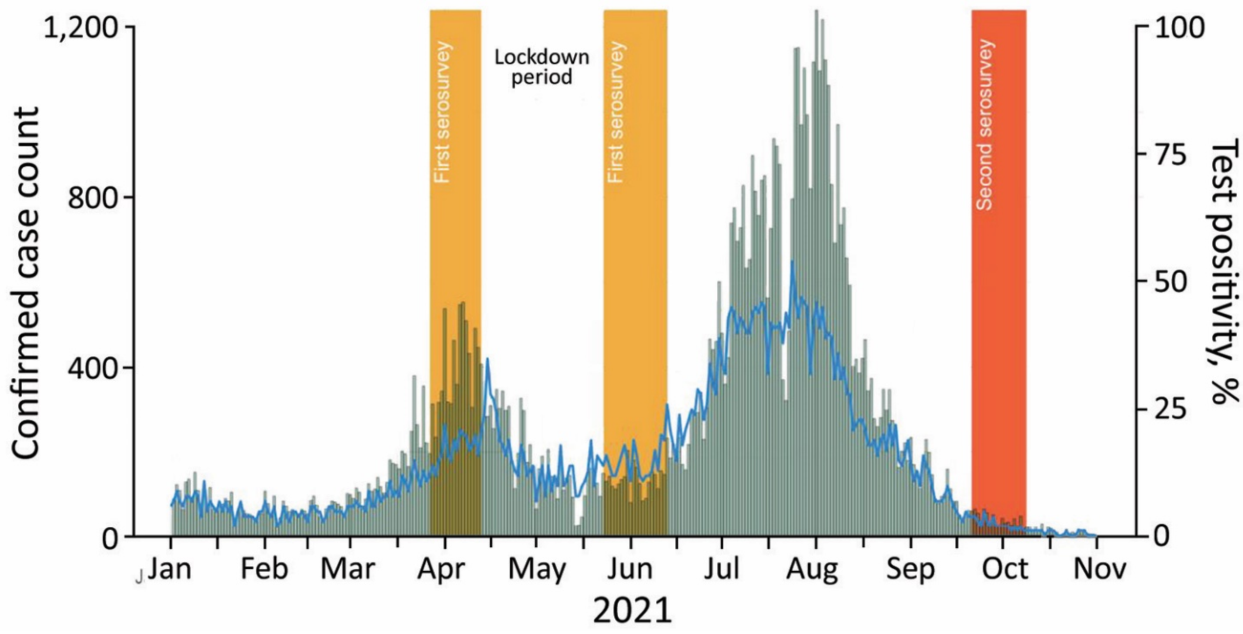

1,200
800
400
0
Confirmed case count
First serosurvey
Lockdown period
First serosurvey
Second serosurvey
100
75
50
25
0
Test positivity, %
Jan
Feb
Mar
Apr
May
Jun
Jul
Aug
Sep
Oct
Nov
2021
